# Citral induced apoptosis in MDA-MB-231 spheroid cells

**DOI:** 10.1186/s12906-018-2115-y

**Published:** 2018-02-13

**Authors:** Siyamak Ebrahimi Nigjeh, Swee Keong Yeap, Norshariza Nordin, Behnam Kamalideghan, Huynh Ky, Rozita Rosli

**Affiliations:** 1 Institute of Bioscience, Universiti Putra Malaysia, 43400 Serdang, Selangor Malaysia; 2 China-ASEAN College of Marine Sciences, Xiamen University Malaysia, Jalan Sunsuria, Bandar Sunsuria, 43900 Sepang, Selangor Darul Ehsan Malaysia; 3 Faculty of Medicine and Health Sciences, Universiti Putra Malaysia, 43400 Serdang, Selangor Malaysia; 4 Medical Genetics Department, School of Medicine, Shahid Beheshti University of Medical Sciences, Tehran, 1983963113 Iran; 5 Department of Agriculture Genetics and Breeding, College of Agriculture and Applied Biology, Cantho University, Cantho, 84071 Vietnam

**Keywords:** Citral, Wnt, Spheroids, ALDH1

## Abstract

**Background:**

Breast cancer remains a leading cause of death in women worldwide. Although breast cancer therapies have greatly advanced in recent years, many patients still develop tumour recurrence and metastasis, and eventually succumb to the disease due to chemoresistance. Citral has been reported to show cytotoxic effect on various cancer cell lines. However, the potential of citral to specifically target the drug resistant breast cancer cells has not yet been tested, which was the focus of our current study.

**Methods:**

The cytotoxic activity of citral was first tested on MDA-MB-231 cells in vitro by MTT assay. Subsequently, spheroids of MDA-MB-231 breast cancer cells were developed and treated with citral at different concentrations. Doxorubicin, cisplatin and tamoxifen were used as positive controls to evaluate the drug resistance phenotype of MDA-MB-231 spheroids. In addition, apoptosis study was performed using AnnexinV/7AAD flowcytometry. Aldefluor assay was also carried out to examine whether citral could inhibit the ALDH-positive population, while the potential mechanism of the effect of citral was carried out by using quantitative real time- PCR followed by western blotting analysis.

**Results:**

Citral was able to inhibit the growth of the MDA-MB-231 spheroids when compared to a monolayer culture of MDA-MB-231 cells at a lower IC_50_ value. To confirm the inhibition of spheroid self-renewal capacity, the primary spheroids were then cultured to additional passages in the absence of citral. A significant reduction in the number of secondary spheroids were formed, suggesting the reduction of self-renewal capacity of these aldehyde dehydrogenase positive (ALDH^+^) drug resistant spheroids. Moreover, the AnnexinV/7AAD results demonstrated that citral induced both early and late apoptotic changes in a dose-dependent manner compared to the vehicle control. Furthermore, citral treated spheroids showed lower cell renewal capacity compared to the vehicle control spheroids in the mammosphere formation assay. Gene expression studies using quantitative real time PCR and Western blotting assays showed that citral was able to suppress the self-renewal capacity of spheroids and downregulate the Wnt/β-catenin pathway.

**Conclusion:**

The results suggest that citral could be a potential new agent which can eliminate drug-resistant breast cancer cells in a spheroid model via inducing apoptosis.

## Background

Breast cancer remains the second leading cause of death in women worldwide. Recent advances in the treatment modalities including endocrine therapy have greatly improved the survival of breast cancer patients [1]. Advances in research in the areas of many natural or synthetic phytochemicals documented their ability to inhibit the growth of tumour cells both in vitro and in vivo. Many of these compounds have anti-oxidant, anti-proliferative and pro-apoptotic effects on a number of cancers [2]. Among the many natural agents, Citral (3,7-dimethyl-2,6-octadien-1-al), an aldehyde component in the essential oil extracted from citrus fruits, lemongrass and ginger, has been shown to have anti-proliferative and pro-apoptotic activity in MCF-7 and MDA-MB-231 breast cancer cell lines [3]. Furthermore, an in vivo study showed anti-tumor activity of citral on 4 T1 breast cancer xenograft mouse model [4]. The focus of the current research was to study the potential of citral to specifically target drug resistant breast cancer cells, which have yet to be tested.

Although there are many treatment modalities for breast cancer patients, many patients still suffer from tumour recurrence and metastasis and eventually succumb to this disease due to both intrinsic (*denovo*) and extrinsic (acquired) chemoresistance, which clearly calls for the identification of novel therapeutic agents that could kill drug resistant breast cancer cells. The recent discovery of cancer cell subclones with Aldehyde dehydrogenase 1 (ALDH1) activity contributed to the poor prognosis of breast cancer [5], opens newer areas of research. ALDH1A1 was identified as a putative enzyme that converts retinol to retinoic acid in differentiation pathways of normal and cancer stem cells. The ALDH1A1 subtype of breast cancer stem cells has been found to be responsible for resistance to chemotherapeutic drugs [6]. Thus, targeted suppression of ALDH1A1 activity may help to sensitize breast cancer cells to traditional cancer therapy. Other cell signaling pathways may also contribute to the drug-resistant phenotype and tumour progression of breast cancer, especially because silencing of ALDH expression was not sufficient to fully overcome drug-resistance phenotype of breast cancer cells [6]. Besides ALDH activity, Wnt protein has also been suggested to play an important regulatory role in tumour progression in breast cancer, further suggesting that the Wnt/β-catenin pathway could be a potential therapeutic target in overcoming drug-resistance in breast cancer [7].

In vitro tumor spheroids is a method to culture cancer cells in 3-dimensional form. A previous study showed that breast cancer spheroids were resistant to tamoxifen [8]. This drug-resistant characteristics of breast cancer spheroids is in part contributed by the overexpression of ALDH1 [9] and Wnt proteins [10, 11]. Thus, the cancer spheroids model has been utilized to evaluate the potential activity of phytochemical sulforaphane isolated from broccoli sprouts [12]. Sulforaphane targeted drug-resistant human breast cancer spheroids by decreasing the ALDH1 population and downregulating Wnt/beta-catenin self-renewal pathway [12]. Although anti-tumor effect of citral has been reported previously in breast cancer cell lines [3, 4], the regulation of citral on self-renewal capacity of drug resistant breast cancer cells has not yet been tested. Therefore, our current study was aimed at evaluating the regulatory role of citral on ALDH1 and Wnt pathways using drug resistant spheroid model of MDA-MB-231 cells. Our results suggest that citral could function as a novel preventive and therapeutic agent for the management of breast cancer by eliminating drug-resistant cells which will likely lead to overcoming tumour recurrence and metastasis toward improving the overall survival of breast cancer patients.

## Methods

### Cell line and reagents

The breast cancer cell line MDA-MB-231 was obtained from the American Type Culture Collection (ATCC). Citral (Geranial and neral mixture, cat no: C83007) was purchased from Sigma Aldrich, USA. The stock solution was prepared in 5% dimethyl sulfoxide (DMSO) (Sigma Aldrich, USA) and stored at − 20 °C until further use. Antibodies to β-catenin, phospho-β-catenin Ser33/Ser37/Thr41 were purchased from Cell Signaling Technology, USA. Antibodies to cyclin D1 and β-actin were purchased from Santa Cruz Biotechnology, USA.

### Monolayer MTT cell viability assay

MDA-MB-231 cells were seeded into each well of a 96-well microculture plate (TPP, Switzerland) at a concentration of 2 × 10^5^ cells/mL. Simultaneously, cells in another 96-well microculture plate were treated with various concentrations of citral (30 μg/mL as highest concentration). After 48 h of incubation, cell viability was assessed by MTT assay (Promega, USA) according to the manufacturer’s instructions.

### Spheroid formation and Citral treatment

Spheroids of breast cancer cells were developed and cultured as previously described [13] using a serum-free mammary epithelium basal medium (Gibco, USA). After 7 days of culture, the numbers and morphology of spheroids were observed using a Nikon Eclipse light microscope. Then, the MDA-MB-231 cells-derived spheroids were treated with citral at different concentrations (2.5, 5.0 and 10.0 μg/mL) for 48 h. After the treatment period, vehicle control and citral treated spheroids were observed under Nikon Eclipse light microscope (the image of 4 random areas per well were taken for each group, and the experiment was carried out in triplicate) and the average volume of the spheroid was calculated using V = (4/3) π R^3^. (*n* = 12 images per group). The harvested spheroids were also dissociated into single cells by incubating the spheroids in enzymatic solution using 0.25% trypsin-EDTA (Gibco USA) for 30 min followed by mechanical dissociation using pipetting. The harvested single cells suspension were subjected to the following assays.

### MTT assay for doxorubicin, Cisplatin and Tamoxifen on MDA-MB-231 spheroids

Cisplatin, doxorubicin and tamoxifen were used as positive control to evaluate the drug resistance phenotype of MDA-MB-231 spheroids. Briefly, the MTT solution (Sigma, USA) was dissolved in phosphate buffered saline (PBS) at 5 mg/mL. Twenty μL of 5 μg/mL MTT solution was added directly to all appropriate wells. Cells were plated in 96-well plates at an initial density of 1 × 10^5^ cells/mL. After incubation for 24 h at 37 °C, cells were treated with various concentrations of doxorubicin, cisplatin and tamoxifen and incubated for 24, 48 and 72 h. The MTT solution was added to each well and further incubated for 4 h at 37 °C. The optical density was read with an enzyme-linked immunosorbent assay (ELISA) reader (Bio-Tek Instruments, USA) at 570 nm. Each concentration of drugs was assayed in triplicate. The percent cell viability was calculated as follows:

$$ \mathrm{Cell}\ \mathrm{viability}\ \left(\%\right)=\frac{\kern0.75em \mathrm{OD}\ \mathrm{of}\ \mathrm{Teatment}\ }{\mathrm{OD}\kern0.5em \mathrm{of}\ \mathrm{Control}}\times 100 $$

IC_50_ values (Table 1) were determined by plotting a linear regression curve.

### Effect of Citral on MDA-MB-231 spheroid formation assay and cell-renewal

In ultra-low attachment plates, primary spheroid cultures derived from MDA-MB-231 cell line were treated with different concentrations (2.5, 5 and 10 μg/mL) of citral (Sigma-Aldrich, USA). The spheroid formation was measured after 7 days of treatment using a Nikon Eclipse microscope (Nikon, Japan). Then, single cells from untreated and treated primary spheroid cultures were obtained by incubation in a 0.25% trypsin-EDTA solution (Gibco. USA), for about 5–10 min at 37 °C. Subsequently, the cells were plated in ultra-low attachment plates at a density of 100 to 500 cells/mL for the generation of subsequent passages. The second passage was grown in the absence of treatment. The spheroids were observed after 7 days using a Nikon Eclipse microscope (Nikon, Japan).

### AnnexinV/7AAD apoptosis assay

AnnexinV/7AAD assay was performed on the control and citral treated MDA-MB-231 spheroids according to the manufacturer’s protocol (FITC Annexin V Apoptosis Detection Kit with 7-AAD, BioLegend USA. Catalog no: 640,242). Briefly, the cells were washed twice with cold BioLegend’s cell staining buffer and then resuspended in Annexin V Binding Buffer at a concentration of 1.0 × 10^7^ cells/mL. The cells were then mixed and 100 μL of the cell suspension was transferred to a 5 mL test tube, followed by the addition of 5 μL of FITC Annexin V, and 5 μL of 7-AAD Viability Staining Solution to the tube. The cells were gently vortexed and incubated for 15 min at room temperature (25 °C) in the dark. Then, 400 μL of Annexin V Binding Buffer were added to each tube, following which the samples were analyzed by flowcytometry (BD FACSCalibur, USA). Each sample was tested in triplicates.

### Aldefluor assay

Aldefluor assay was carried out using vehicle control and citral treated MDA-MB-231 spheroids according to the manufacturer’s guidelines (StemCell Technologies, Canada). Single cells obtained from spheroids were incubated in the Aldefluor assay buffer containing bodipyamino-acetaldehyde (1 μmol/L per 10^6^ cells) and ALDH substrate, for 40 to 50 min at 37 °C. A collection of cells from each sample that were incubated under identical conditions in the presence of the ALDH inhibitor diethylaminobenzalde-Hyde was used as a negative control in this assay.

### Quantitative real time-polymerase chain reaction (qRT-PCR)

Total RNA was extracted using RNeasy Mini Kit (Qiagen, USA) according to the manufacturer’s specifications. RNA concentration was quantified using the NanoDrop 2000 Spectrophotometer (NanoDrop Technologies, Thermo, USA). cDNA Synthesis Kit (Ferementas) was used to convert the RNA to cDNA according the manufacturer’s instruction. LifeTech 2 step RT-PCR SYBR Green kit (LifeTech, USA) was used to prepare the reaction mix according to instructions of the manufacturer. The primers (Table 1) for target (adenomatous polyposis coli {APC}, AXIN, Casein kinase 1 {CK1} and Wnt coreceptor low density lipoprotein receptor-related protein 6 {LRP6}) and house keeping (GAPDH, 18 s rRNA, β-actin) genes were designed in a manner to be able to amplify only the cDNAs compatible to mRNA sequence but not any genomic sequences. Gradient PCR, standard curve, and quantitative gene transcription analyses were carried out using the CFX Manager software, version 1.6 (BioRad Laboratories, Inc., Hercules, CA), provided with the real-time PCR thermal cycler (CFX96, BioRad Laboratories, Inc., Hercules, CA).

**Table 1:** Primer sequences in one-step SYBR green quantitative real time PCR

Accession Number	Name	Sequence (5’➔ 3′)
NM_001271741.1	CK1 F	GAGATCCCTTTCCCAGAGTGC
	CK1 R	TTTGTGAAGGGCTTCTCGGC
NM_000038.5	APC1 F	AGCAAGTTGAGGCACTGAAGA
	APC1 R	TCCCGGCTTCCATAAGAACG
XM_005255610.2	AXIN F	TTTCACCGAAGATGCTCCCC
	AXIN R	CACTGCCCTCAGGCTCATAC
NM_002336.2	LRP6 F	ACATGACAGGTCGAGAGGGT
	LRP6 R	CCAAGCCACAGGGATACAGT
NM_001101.3	β-actin F	CCAGACTCATTCAACCAGACA
	β-actin R	GATGACTGAGTACCTGAACCG
NM_002046.4	GAPDH F	CGGGACCTAATGAAACTCCA
	GAPDH R	AATCTCCACTTTGCCACTGC
HQ387008.1	18S rRNA F	GTAACCCGTTGAACCCCATT
	18S rRNA R	CCATCCAATCGGTAGTAGCG

### Western blotting analysis

In this study, Western blotting was used to determine the expression of β-catenin-93 KDa, CyclinD1–33 KDa, Phospho β-catenin 33/337–50 KDa, protein in cell lysates. β-actin 42 KDa was selected as the reference protein. The antibody-antigen complexes were subsequently identified by horseradish peroxidise (HRP). The blot was visualized using chemiluminescent detection substrate, and we used Chemiluminescence ONE-HOUR WesternTM Advanced Kit. The membrane was quantified using a Biospectrum AC ChemiHR 40 system (UVP, Upland, CA, USA). ImageJ software was used to quantify the band intensity for all the targeted proteins. Relative expression analysed of β-catenin, Cyclin D1 and Phospho 33/337 between vehicle control and citral treated cells were calculated after normalizing to the intensity of beta actin for each group.

### Statistical analysis

All the experiments were performed in three biological replicates and each with triplicates. Results were expressed as mean ± SD. Statistical analysis was done using SPSS version 17.0 (SPSS Inc., Chicago, USA). Probability values of less than alpha 0.05 (*P <* 0.05) were considered statistically significant.

## Results and discussion

### Cytotoxicity of citral on MDA-MB-231 breast cancer cells

**Fig. 1:**
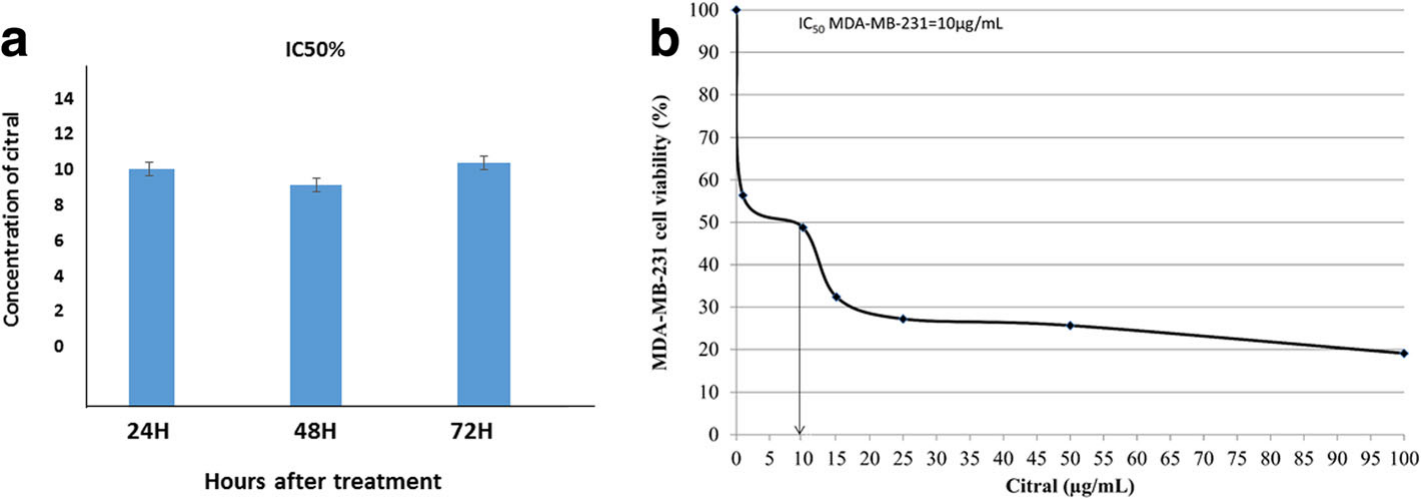
Viability of cital treated MDA-MB-231 cells for 24,48 and 72 h (**a**). The IC_50_% of citral after 48 h treatment on MDA-MB-231 cells (**b**). All the experiments were performed in three biological replicates and each with triplicates. All data are expressed as mean ± S.E.M. with (*p < 0.05)

The cytotoxic effect of citral in MDA-MB-231 cells was evaluated by MTT assay. The percentage of viable treated cells relative to vehicle control was calculated. Survival of MDA-MB-231 cells was decreased as the concentration of citral increased with IC_50_ (concentration of citral that reduced 50% of cell viability relative to vehicle control) was 10.00 ± 0.14 μg/mL for MDA-MB-231 cells at 48 h incubations (Fig. 1).

### Morphological characterization by light microscopy

**Fig. 2:**
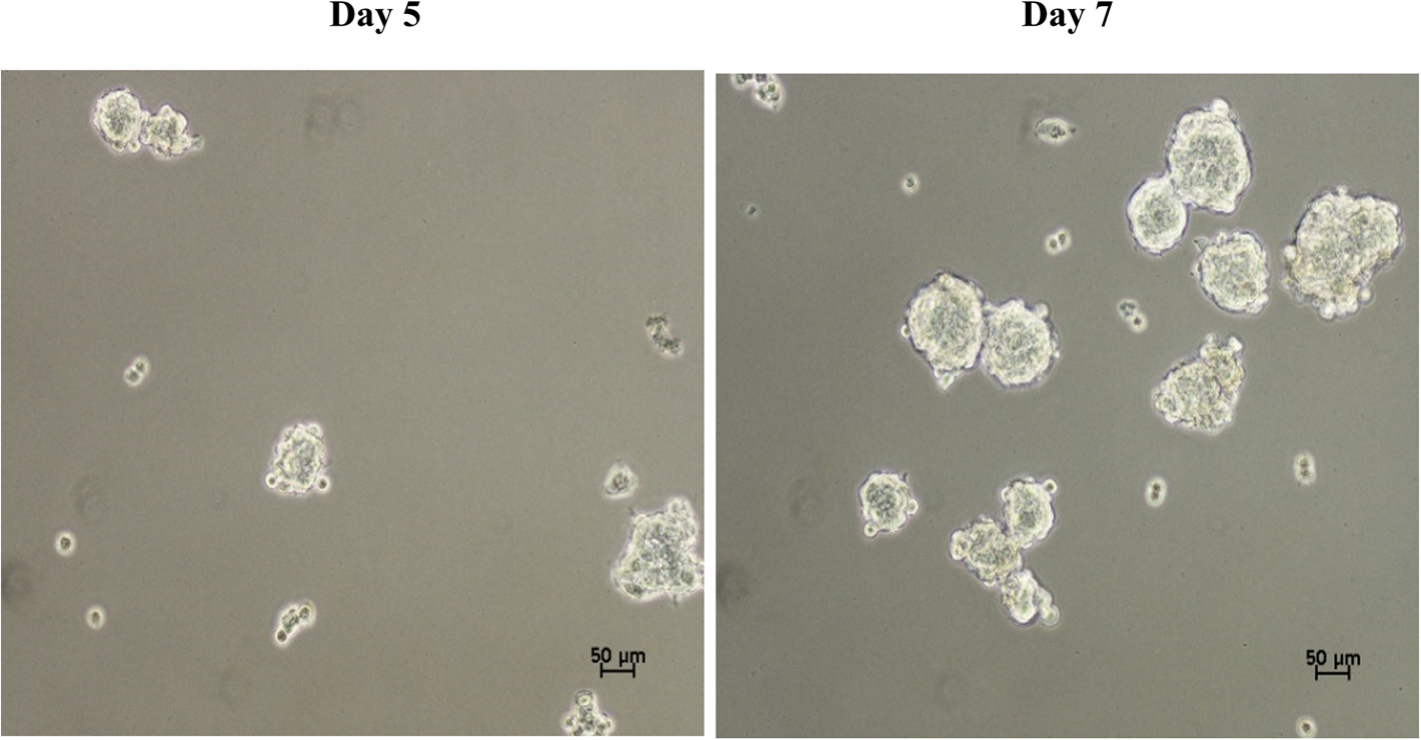
Spheroid formation of MDA-MB-231 in serum free media at (**a**) day 5 and (**b**) day 7. All the experiments were performed in three biological replicates and each with triplicates. All data are expressed as mean ± S.E.M. with (*p < 0.05)

**Fig. 3:**
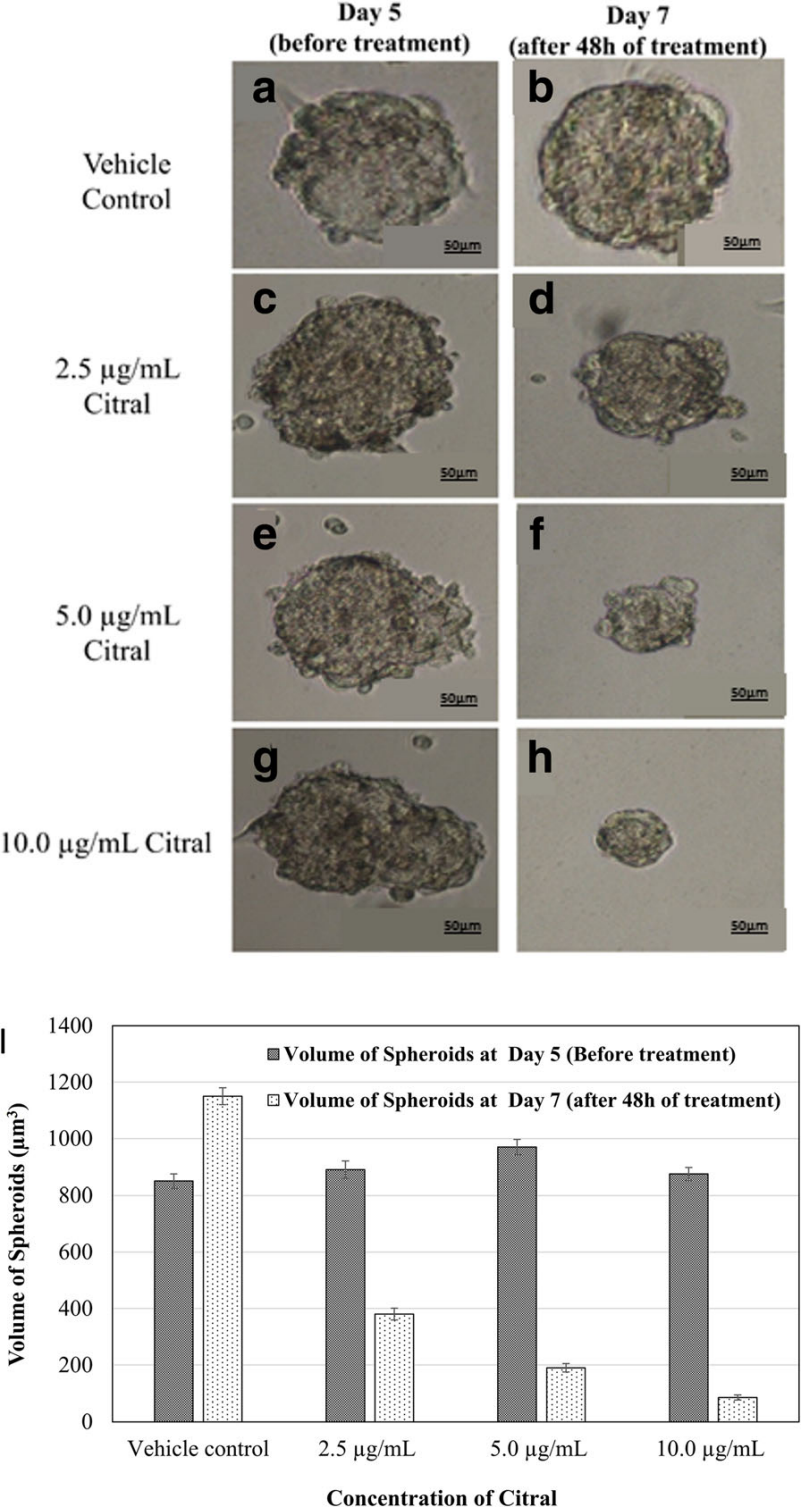
**a-h** MDA-MB-231 spheroid volume before (day 5) and after (day 7) citral treatment (magnification, × 100). **i** Volume quantification of vehicle control and citral treated MDA-MB-231 spheroid. The volume of MDA-MB-231 spheroid was estimated using V = (4/3) π R^3^. All the experiments were performed in three biological replicates and each with triplicates. All data are expressed as mean ± S.E.M. with (*p < 0.05)

MDA-MB-231 cells were capable of forming spheroids in serum-free culture medium. The spheroids appeared on day 5 (Fig. 2a), with increased diameter at day 7 (Fig. 2b). The IC_50_ value of tamoxifen on MDA-MB-231 spheroids was 2-fold higher (~ 16 μg/mL) than the monolayer culture of MDA-MB-231 cells (~ 8 μg/mL) after subsequent 48 h (result not shown) of incubation indicating the drug resistant behaviour of the spheroids compared to the monolayer culture. After 5 days of culture, the MDA-MB-231 spheroids were treated with various concentrations of citral. Citral decreased the size (Fig. 3a-h) and volume (Fig. 3i) of primary spheres formed. The spheroids size reduction is related to decreasing proliferation and also induction of apoptosis, which is confirmed by Annexin V/7AAD flowcytometry and measuring the level of Cyclin D1 using western blot (Table 2).

**Table 2:** IC_50_ values of doxorubicin, cisplatin, tamoxifen and citral on MDA-MB-231 monolayer and spheroid

	MDA-MB-231 monolayer	MDA-MB-231 spheroid
Doxorubicin (μg/mL)	0.41 ± 0.23	4.96 ± 1.81
Cisplatin (μg/mL)	1.72 ± 0.31	8.52 ± 2.13
Tamoxifen (μg/mL)	8.35 ± 1.41	16 ± 2.58

### Effect of Citral on MDA-MB-231 spheroid formation assay and cell-renewal

**Fig. 4:**
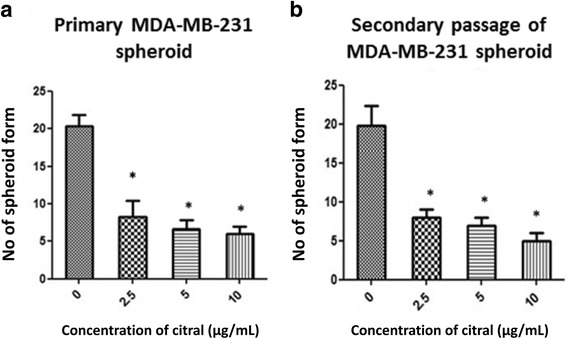
Inhibitory effects of citral on MDA-MB-231 spheroid (**a**) first passage and (**b**) second passage (in the absence of citral) comparison with vehicle control. All the experiments were performed in three biological replicates and each with triplicates. All data are expressed as mean ± S.E.M. with (*p < 0.05)

To confirm the inhibition of spheroid self-renewal capacity by citral, the vehicle control and citral treated spheroids were then cultured to additional passages in the absence of citral. As shown in Fig. 4a, citral caused inhibition on the formation of MDA-MB-231 primary spheroids, where the number of spheroids declined from 20 spheroids in untreated group to 8, 7 and 6 spheroids when treated with citral at concentrations of 2.5, 5.0 and 10.0 μg/mL. To confirm the inhibition of spheroid self-renewal capacity, the treated spheroids (primary spheroids) were then cultured to additional passages in the absence of citral. The number of spheroids at secondary passage without citral treatment at day 5 were still inhihbited as observed in the primary culture, suggesting the reduction of self-renewal capacity of these ALDH^+^ drug-resistant spheroids (Fig. 4a and b). In the absence of citral, the second passage that were derived from citral-treated primary spheroids yielded a slightly lower number of spheroids in comparison with the vehicle control for MDA-MB-231 spheroids (Fig. 4b). As shown in Fig. 4b, the number of secondary spheroids derived from primary spheroids treated at different concentrations of citral (2.5, 5.0 and 10.0 μg/mL) were still inhibited at 8, 7 and 5 spheroids, respectively (*P* < 0.01). More interesting, the concentrations of citral that were capable of suppressing MDA-MB-231 spheroid formation (IC_50_, 2.5–5.0 μg/mL) were 2-folds lower than those exhibited in the MTT assay (IC_50_, 10.0 μg/mL). The reduction remained in the number of secondary sphere-forming capacity which suggests a reduction of self-renewal capacity of these drug-resistant spheroids (Fig. 4a and b).

### Annexin V/7AAD Flowcytometry

**Fig. 5:**
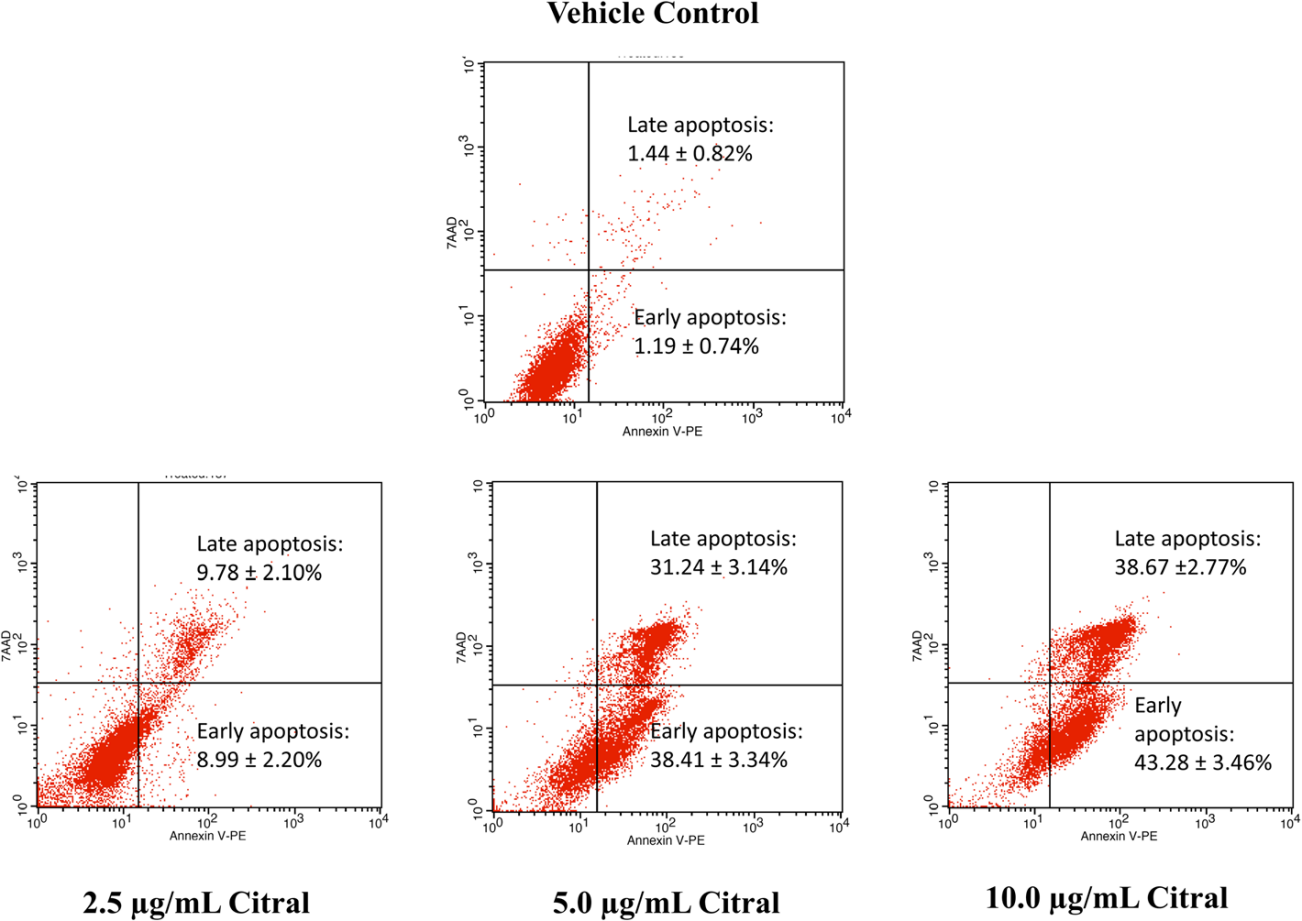
Annexin V/7AAD flowcytometry. MDA-MB-231 spheroids with low concentration (2.5 μg/mL) of citral, showed 8.99 ± 2.20% and 9.78 ± 2.10% for early and late apoptosis respectively, increasing the concentration to 5.0 μg/mL showed 38.41 ± 3.34% and 31.24 ± 3.14% and the high concentration (10.0 μg/mL) showed 43.28 ± 3.46% and 38.67 ± 2.77% for early and late apoptosis respectively compared to vehicle control that showed 1.19 ± 0.74% and 1.44 ± 0.82% of early and late apoptosis. All the experiments were performed in three biological replicates and each with triplicates. All data are expressed as mean ± S.E.M. with (*p < 0.05)

The apoptosis and cell membrane integrity were determined in the present study using BioLegend FITC Annexin V Apoptosis Detection Kit with 7-AAD by flow cytometry. By using single-laser multiparameter flow cytometry on unfixed cells, live cells could be distinguished from early and late stage apoptotic cells stained with multicolor FITC 7-AAD and Annexin V-PE dyes. Figure 5 shows a significant difference (*p* < 0.001) in early and late apoptosis of treated MDA-MB-231 spheroids and vehicle control. The spheroids were exposed to citral at different concentrations, 2.5 μg/mL and 5.0 μg/mL and 10.0 μg/mL and compared to the vehicle control. The vehicle control showed 1.19 ± 0.74% and 1.44 ± 0.82% of early and late apoptosis, respectively while incubation of MDA-MB-231 spheroids with low concentration (2.5 μg/mL) of citral increased the early and late apoptosis to 8.99 ± 2.20% and 9.78 ± 2.10%, respectively. The percentage of early and late apoptosic cells were increased to 38.41 ± 3.34% and 31.24 ± 3.14% with the concentration of citral at 5.0 μg/mL.. In comparison, the high concentration of citral (10.0 μg/mL) showed 43.28 ± 3.46% and 38.67 ± 2.77% of early and late apoptosis, respectively. Therefore, citral at all three concentrations exhibited apoptotic effect on MDA-MB-231 spheroids compared to the vehicle control.

### Aldefluor assay

**Fig. 6:**
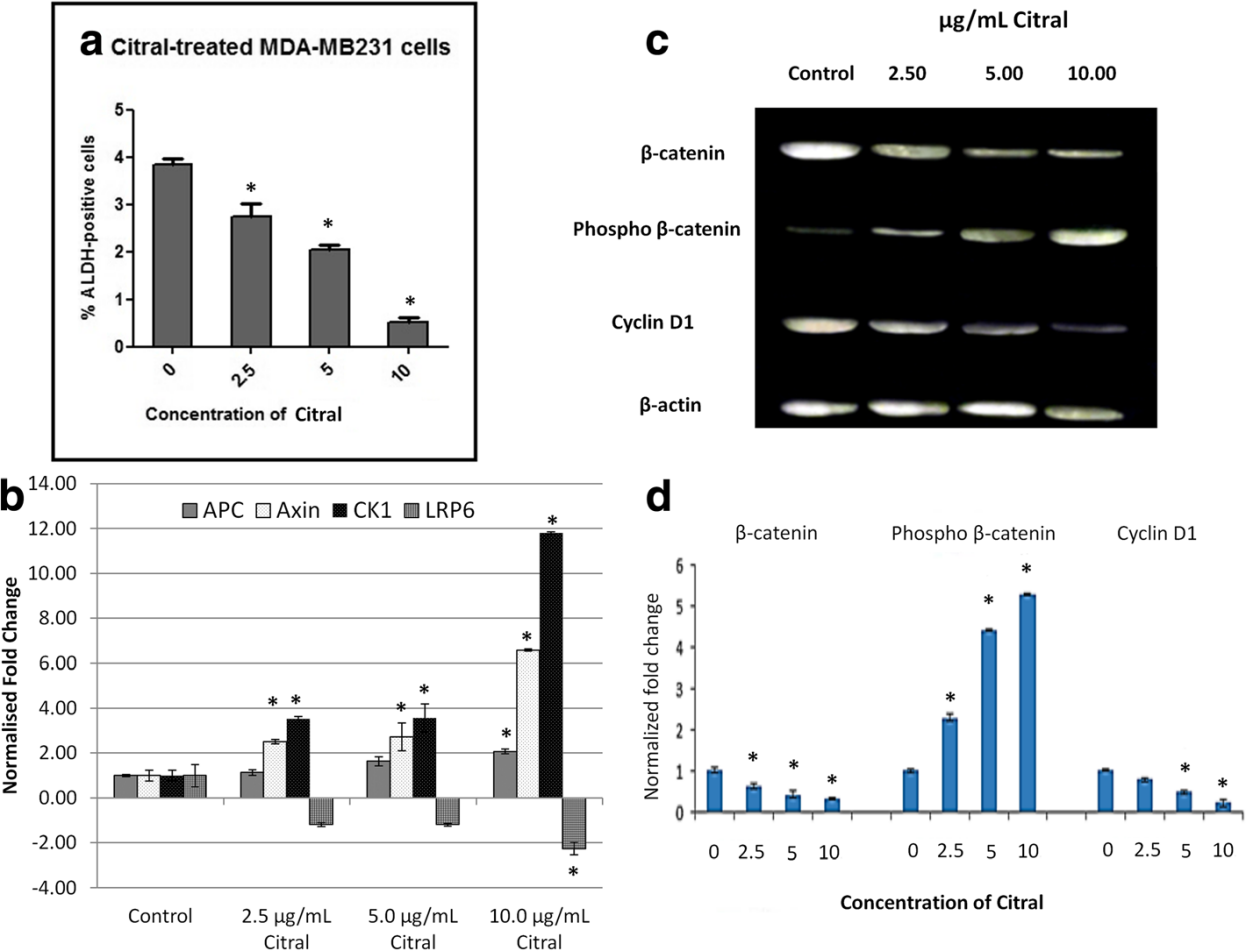
**a** Inhibitory effect of citral on ALDH positive-cell population. The MDA-MB-231 spheroids were treated with citral (2.5, 5 and 10 μg/mL) and subject to Aldefluor assay flow cytometry analysis. Citral decreased the percentage of ALDH-positive cells. **b** Relative expression level of APC, AXIN, CK1 and LRP6 in vehicle control and citral treated MDA-MB-231 spheroids. **c** Representative blot of β-catenin, phospho β-catenin and cycline D1 protein level in vehicle control and citral treated MDA-MB-231 spheroids. **d** Quantification of western blot analysis for β-catenin, phospho β-catenin and cycline D1 protein level in vehicle control and citral treated MDA-MB-231 spheroid using ImageJ software. All the experiments were performed in three biological replicates and each with triplicates. All data are expressed as mean ± S.E.M. with (*p < 0.05)

To examine whether citral could inhibit the ALDH-positive cells in vitro, MDA-MB-231 spheroids was treated with the same concentration of citral. Citral significantly decreased the ALDH-positive population of MDA-MB-231 spheroids in a dosage dependent manner (Fig. 6a).

### Quantitative real-time PCR and western blot analyses

The Wnt/β-catenin pathway is an important regulatory pathway of stem cell self-renewal. Hence, the regulation of β-catenin and Wnt/β-catenin downstream targets were examined after citral treatment. To quantify the regulation of Wnt pathway in MDA-MB-231 spheroids, quantitative real time-polymerase chain reaction was performed to evaluate the differential mRNA expression of APC, AXIN, CK1 and LRP6 (normalized to the Glyceraldehyde 3-phosphate dehydrogenase (GAPDH), β-actin and 18 s rRNA) between the vehicle control and citral treated spheroids. We found that APC, Axin and CK1 were upregulated by citral treatment with increasing concentrations (2.5, 5 and 10 μg/mL) compared to the vehicle control. Whereas, LRP6 was downregulated by citral treatment in a dosage dependent manner (Fig. 6b).

Furthermore, regulation on Wnt/β-catenin pathway in MDA-MB-231 spheroids by citral was further confirmed by evaluating the protein levels of β-catenin, phosphor-β-catenin and cyclin D1 by western blot analysis. As shown in Fig. 6c, citral treatment led to decreased protein levels of β-catenin and cyclin D1 while the levels of phospho β-catenin in MDA-MB-231 spheroids was increased in dosage dependent manner (Fig. 6c and d).

## Discussion

According to Cravotoo et al.*,* [14], citral was rapidly absorbed from the gastro-intestinal tract of mouse and rat, and also much of an applied dermal dose was lost due to its extreme volatility, but the citral remaining on the skin was fairly well absorbed. Besides that, citral was rapidly metabolized and excreted as metabolites and urine is the major route of elimination. Acute toxicity of this chemical is low in rodents because the oral or dermal lethal dose (LD_50_) values were more than 1000 mg/kg, this chemical is irritating to skin and not irritating to eyes in rabbits [15]. Citral has been previously reported to exhibit cytotoxic activity against breast [3] and hematopoietic [16] cancer cell lines through the induction of apoptosis. Similarly, our data has shown that the IC_50_ value on MDA-MB-231 cells is 10 μg/mL (Fig. 1a). However, the potential of citral to specifically target the drug resistant breast cancer cells has not yet been tested which was the focus of our current study. Ricardo et al. [17] demonstrated that drug resistant breast cancer cells, which contained higher ALDH1 activity survived and formed spheroids when cultured in serum-free medium. Furthermore, a previous study has shown that the ability of spheroids to be consecutively passaged is an indirect marker of drug resistant cancer cell’s self-renewal capacity [13]. Thus, MDA-MB-231 spheroids were used as an in vitro culture model (Figs. 2, 3 and 4) to evaluate the cytotoxicity of citral on drug resistant breast cancer cells in this study. The cultured MDA-MB-231 spheroids showed higher levels of ALDH1 activity (Fig. 6a), which underwent self-renewal (indicated by the capacity of sphere formation in subsequent passages (Fig. 4b), and also showed higher IC_50_ value against tamoxifen (results not shown). The MDA-MB-231 spheroids treated with citral at different concentrations (2.5 μg/mL, 5.0 μg/mL and 10.0 μg/mL) showed more than 7 and 30 fold increase at early and late apoptotic populations, respectively when compared to the vehicle control. The connection between Wnt/β catenin signaling pathway, ALDH drug resistant population and apoptosis is been well established, but different studies have shown that Wnt signaling regulates early and late stages apoptosis through a selection of mechanisms. Moreover, the expression of cyclin D1 following DNA damage is essential for cell cycle re-entry and apoptosis [18–21]. MDA-MB-231 spheroids treated with different concentrations of citral (Fig. 6a) demonstrated significantly lower ALDH+ population, poorer sphere formation efficiency (both primary and secondary passages of MDA-MB-231 spheroids), consistent with reduced volume of the treated spheroids in a dosage dependent manner.

As we observed that citral can effectively control the self-renewal of breast cancer spheroids, the effects of citral in regulating Wnt/β-catenin signaling pathway of MDA-MB-231 spheroids was further investigated with western blot and quantitative real time PCR analyses. β-catenin is the key effector of Wnt signaling pathway, which regulates multiple important biological processes such as cell proliferation and stem cell maintenance [22]. Axin and APC are the tumor suppressor genes that bind β-catenin and recruit CK1 to facilitate destruction of β-catenin through phosphorylation [23]. Moreover, cancer incidences are always found to be associated with dyregulation of Wnt signaling pathway [24]. In addition to that, overexpression of β-catenin and Wnt pathway targeted gene, cyclin D1 were found to serve as poor prognostic markers in human cancer especially in breast cancer [25]. Hence, hyperactive transcription of Wnt signaling and downregulation of Wnt signaling related tumor suppressor genes such as APC, indicate higher levels of self-renewal and dyregulated proliferation of these cancer cells [22, 26]. A previous review has shown that drug targeting on aberrant Wnt signaling pathway can favour cancer treatment outcome [27]. For example, Niclosamide that blocked Wnt co-receptor LRP6 suppressed the growth of Wnt-driven MDA-MB-231 and T-47D breast cancer cells [28]. In this study, we found that mRNA expression of Wnt destruction complex Axin, APC and CK1 were upregulated, while Wnt co-receptor LRP6 was downregulated in MDA-MB-231 spheroids by citral treatment in a dosage dependent manner. These activities are may lead to the phosphorylation of β-catenin and target the phospho β-catenin to proteosomal degradation, which essentially suppressed the Wnt/β-catenin signaling pathway and expression of its target gene, cyclin D1. These results have proposed that citral treatment may regulate the growth of MDA-MB-231 spheroids through downregulation of Wnt signaling and its downstream cell cycle target gene, cyclin D1. However, further studies must be performed to validate the interaction of citral treatment with the components of the Wnt signaling pathway.

## Conclusions

This pilot proof-of concept study provided compelling evidence in support of the anti-tumor activity of citral, a mixture of geranial and neral isomers, whose action is in part mediated through the induction of apoptosis, elimination of spheroids and its self-renewal capacity. This property shall be further compared between different isomers of citral. In addition, future studies using long term drug resistant variants of MDA-MB-231 cells and in vivo xenograft models using these drug resistant cells shall be performed to further support the potential of citral as an alternative treatment to overcome drug resistant breast cancer.

## Abbreviations

ALDH: Aldehyde dehydrogenases
APC: *Adenomatous polyposis coli*
CK1: Casein kinase 1
LRP6: Low-density lipoprotein receptor related protein 6.
MDA-MB-231: Human breast adenocarcinoma
